# Anti-Inflammatory Properties, Bioaccessibility and Intestinal Absorption of Sea Fennel (*Crithmum maritimum*) Extract Encapsulated in Soy Phosphatidylcholine Liposomes

**DOI:** 10.3390/nu14010210

**Published:** 2022-01-04

**Authors:** Ailén Alemán, Daniel Marín-Peñalver, Pilar Fernández de Palencia, María del Carmen Gómez-Guillén, Pilar Montero

**Affiliations:** Institute of Food Science, Technology and Nutrition (ICTAN-CSIC), Ciudad Universitaria, 28040 Madrid, Spain; dmarin@ictan.csic.es (D.M.-P.); pfpalencia@ictan.csic.es (P.F.d.P.); cgomez@ictan.csic.es (M.d.C.G.-G.)

**Keywords:** sea fennel, phosphatidylcholine liposomes, anti-inflammatory properties, chlorogenic acid, gastrointestinal digestion, intestinal absorption

## Abstract

A sea fennel (*Crithmum maritimum*) aqueous extract was prepared and loaded into soybean phosphatidylcholine liposomes. Both the free extract (FE), and the empty (L) and loaded (L-FE) liposomes were shown to be non-cytotoxic to THP-1 and Caco-2 cells. The anti-inflammatory effect was tested on THP-1 cells differentiated into macrophages. FE showed anti-inflammatory activity, revealed by the induced secretion of IL-10 cytokines in macrophages that were subsequently stimulated with LPS. Also, a decrease in TNF-α production by L was observed, evidencing that liposomes reduced the pro-inflammatory mediators’ secretion. The liposomes (L) showed protective anti-inflammatory activity and also were able to downregulate the inflammation. Furthermore, L-FE were also found to downregulate the inflammation response, as they were able to decrease TNF-α secretion in macrophages previously exposed to LPS. The simulated in vitro gastrointestinal digestion (GID) of FE diminished the chlorogenic acid content (the main polyphenolic compound of the extract) by 40%, while in L-FE, the amount of this phenolic compound increased with respect to the undigested liposomes. The amount of bioaccessible chlorogenic, however, was similar for FE and L-FE. The percentage of chlorogenic acid absorbed through a Caco-2 cell monolayer after 3 h of incubation, was significantly similar for the extract and the liposomes (~1.5%), without finding significant differences once the extract and liposomes were digested.

## 1. Introduction

*Crithmum maritimum,* commonly named sea fennel, is an edible halophile plant that is very abundant on the Mediterranean and Atlantic seacoasts. Sea fennel is especially used in gourmet cuisine, or very locally, being an underutilized plant. In previous work, the polyphenolic composition profile of sea fennel aqueous and ethanolic extracts, and their antioxidant properties were studied [1]. It was observed that its main component was chlorogenic acid, which some studies have related to the antioxidant activity of sea fennel [1,2], as well as to other properties associated with disease prevention, such as anticarcinogenic and antimutagenic activities [2], although there are still few studies on this regard.

Given the high amount of chlorogenic acid present in sea fennel [1,3], to which outstanding antioxidant and anti-inflammatory properties have been attributed [4], sea fennel extract may also have a beneficial effect on inflammation mediators. In this sense, several studies have shown how various polyphenols, including hydroxychinamic acids, increase the activation of the immune system response [5,6]. On the other hand, it has also been observed that phospholipids act as lipidic mediators of inflammation [7]. The use of macrophages is an ex vivo human system, among others, considered adequate and widely used to study the intracellular cytokine expression pathways [8].

Thus, the biotransformation suffered by phenolic compounds during the digestive process and their subsequent intestinal absorption will determine their bioactive effect on the body. The absorption of most phenolic compounds takes place mainly in the small intestine. Most polyphenols are stable to the action of gastric acids [9] therefore neither hydrolysis nor absorption in this phase of the digestive process occurs, allowing them to reach the intestine in their native form. Some phenolic compounds, such as caffeic acid, are absorbed in the stomach owing to their non-ionized form. On the contrary, their esterified analogs, as is the case of chlorogenic acid, usually pass through the stomach [10] and the small intestine intact, most of them reaching the colon, where the colonic microflora probably transforms them on account of their esterase activity [11].

Several studies confirm that the in vitro determination of permeability, through models based on artificial membranes using cell cultures (Caco-2 cells), can help predict the in vivo absorption of compounds [11,12]. In particular, some studies have confirmed an absorption transport for chlorogenic acid, although the percentage of this phenolic compound recovered in the basolateral compartment has been quite low in all cases studied, with values below 3% of the initial amount in the food matrix [11,12,13].

There are various reasons to protect or encapsulate bioactive compounds, some of them are technological, to avoid flavors or interactions with other components in the food matrix, but there are also others, as it allows the possibility to protect the compounds against GID, favoring their intestinal absorption. Different strategies have been conducted in order to improve the intestinal absorption of chlorogenic acid, including its encapsulation in liposomes. According to Li et al. [14], encapsulation of polyphenols can enhance their absorption and bioavailability due to their low solubility as well as to their low stability and permeability in the GI tract, although nanoparticle stability during GID must also be considered. In this sense, Feng et al. [15], managed to increase 1.29 times the bioavailability of chlorogenic acid extract by using soy phosphatidylcholine and cholesterol liposomes (6:1 WW liposome/chlorogenic) in an in vivo study, concluding that encapsulation in liposomes may be a good mechanism to improve the bioavailability of the bioactive compounds which had been encapsulated. The bioavailability of phenolic compounds, in general, is limited in the human body. Manach et al. [16], found in a large review of 97 human trials that, after the intake of 50 mg of total phenol equivalents, a plasmatic concentration of total metabolites between 0 and 4 µM was reached.

In previous work, the encapsulation of two sea fennel extracts (aqueous and ethanolic) in soy phosphatidylcholine liposomes was studied, as well as their most suitable concentrations for good entrapment efficiency and their antioxidant properties [1].

This work aimed to explore the potential anti-inflammatory properties of sea fennel aqueous extract in its free form and encapsulated in liposomes of partially purified soy phosphatidylcholine in order to be considered for food applications. Subsequently, the in vitro bioavailability of both the extract without encapsulation and the extract protected in liposomes was evaluated. In order to do so, the effect of the gastrointestinal digestion process on the phenolic composition of the extract and the filled liposomes was previously determined. Later, the absorption in an intestinal epithelium model was carried out, using bicameral permeable supports with Caco-2 cell monolayers, and evaluating chlorogenic acid as the major phenolic compound in the extract.

## 2. Materials and Methods

### 2.1. Materials and Chemical Reactive

Sea fennel (*Crithmum maritimum*) was kindly provided by Portomuiños S.L. (Cerceda, A Coruña, Spain). Soybean lecithin was purchased from Manuel Riesgo S.A. (Madrid, Spain). The standard of chlorogenic acid was acquired from Sigma-Aldrich (St. Louis, MO, USA). Ethanol, methanol, and acetonitrile were acquired from Panreac Química S.L.U. (Barcelona, Spain).

### 2.2. Preparation of Aqueous Sea Fennel Extract

The sea fennel extract (FE) was prepared in distilled water as described in Alemán et al. [1], heating at 60 °C for 30 min and sonicating (model Q700, Qsonica, CT, USA) at 114 W for 5 min. The dispersion was centrifuged at 9000× *g* rpm (Sorwal RC-5B, Sorwal Instruments, Ramsey, MN, USA) and 4 °C for 15 min. The resulting supernatant was filtered through Whatman No. 1 paper, freeze-dried, and stored at −20 °C.

### 2.3. Preparation of Liposomes

The liposomal dispersion from partially purified soy phosphatidylcholine (L) and the liposomes loaded with the sea fennel extract (L-FE) was prepared as previously described by Alemán et al. [1]. The amount of sea fennel extract selected to load the liposomes was 64% (*w*/*w* with respect to phosphatidylcholine).

The characterization of the liposomes was previously carried out by Alemán et al. [1] where Dynamic Light Scattering analysis of loaded liposomes rendered a z-average of 137.1 ± 0.7 nm, polydispersity index (PDI) of 0.370 ± 0.018, and electronegative Z potential of −27.7 ± 2.8. The entrapment efficiency was 65%.

### 2.4. Cell Viability

Caco-2 cells from human colonic adenocarcinoma and a THP-1 cell line were obtained from the human cell bank at the Centro de Investigaciones Biológicas Margarita Salas (CSIC) (Madrid, Spain). Caco-2 cells (1 × 10^5^/mL) were maintained in Men-Alpha Medium (Invitrogen, Barcelona, Spain) supplemented with 10% (*v*/*v*) heat-inactivated fetal bovine serum (Gibco Life Technologies, Grand Island, NY, USA), and streptomycin (100 µg/mL) and penicillin (100 U/mL) (Gibco Life Technologies) at 37 °C in an atmosphere containing 5% CO_2_. The culture medium was replaced every three days. THP-1 cells (2 × 10^5^/mL) were maintained in RPMI 1640 Medium (Gibco Life Technologies) supplemented as described above and were differentiated into macrophages by adding a final concentration of 25 nM of phorbol 12-myristate13-acetate (PMA) (Sigma–Aldrich, St. Louis, MO, USA) after incubating for 72 h, as described Lund et al. [17]. The cells became adherent and PMA was washed using a pre-warmed RPMI, then they were cultured for 24 h in this medium.

For cell viability, Caco-2 cells were seeded at 10^4^ cells/wells in 100 μL complete culture medium into a 96-well tissue culture plates (Falcon Microtest™, Franklin Lakes, NJ, USA) and incubated to reach 80–90% confluent cells, approximately for two or three days, at 37 °C and 5% CO_2_. THP1- cells, 10^5^ cells/wells in 100 μL complete culture medium, were differentiated into macrophages with PMA, and afterward, they were pre-warmed with 100 μL RPMI as described previously. Then 10 μL of each compound in the two following final concentrations of FE (640, 320 μg/mL), L (1000, 500 μg/mL) and L-FE (1640, 820 μg/mL), were added into the wells against the two cellular lines. They were incubated for 18 h, at 37 °C and 5% CO_2._ Then the supernatant was retired and to test cell viability, both human cell types were treated with 110 μL medium Krebs- Henseleit buffer (Sigma-Aldrich, St. Louis, MO, USA) as a control, or 100 μL Krebs added with 10 μL CCK-8 (Cell Counting Kit-8) solution according to the manufacturer instructions. Afterward, they were incubated for 1–2 h at 37 °C in a dark place (Cell Counting Kit-8, Sigma–Aldrich, St. Louis, MO, USA). Relative cell viability was determined spectrophotometrically at 450 nm by quantification of the formazan dye generated by the activity of dehydrogenases in cells, which is directly proportional to the number of living cells.

### 2.5. Immune Stimulation

To measure the immune response of the THP-1 cells differentiated into macrophages as previously described, 2 × 10^5^ cells/well in 900 μL completed culture medium RPMI were seeded into a 24-well plate. Then 100 μL of the samples at the same final concentrations described above were added and incubated at 37 °C and 5% CO_2_ for 18 h before or after 4 h of stimulation with LPS (O26:B6, 1 μg/mL). A control (untreated and non-stimulated cells) and negative control (untreated and stimulated cells) were obtained. After treatment, samples were centrifuged, 1200× *g* rpm for 5 min, and supernatants were recovered and stored at −20 °C until cytokine analysis. The concentration of each cytokine, IL-10, and TNF-α, released into the supernatants was quantified by using an ELISA kit (Diaclone ELISA Kits, Besancon Cedex, France) according to the manufacturer instructions.

### 2.6. In Vitro Gastrointestinal Digestion

In vitro simulated gastrointestinal digestion of the sea fennel extract (FE) and the liposomes loaded with the extract (L-FE) was carried out according to Alemán et al. [18] with some modifications. Samples were adjusted to pH 2.0 with HCl 1 M, pepsin (EC 232-629-3, Sigma–Aldrich) was added (4% *w*/*w* with respect to FE amount), and the mixture was incubated at 37 °C for 2 h (GD, gastric digestion). The pH was then adjusted to 5.3 with NaHCO_3_ 0.9 M and further to pH 7.5 with NaOH 1 M. After the addition of bile salts (160 mM) and pancreatin (EC 232-468-9, Sigma–Aldrich) (4% *w*/*w* with respect to FE amount), the mixture was incubated at 37 °C for 2 h (GID, gastrointestinal digestion). Digestion was finished by heating at 90 °C for 10 min to inactivate the enzymes. The samples were centrifuged at 13,000× *g* for 30 min, supernatants digested with pepsin (GD) and subsequently digested with pancreatin (GID) were stored at −20 °C until use.

### 2.7. Intestinal Absorption

Caco-2 cells were seeded at a density of 3 × 10^4^ cell/cm^2^ in Transwell inserts with a polycarbonate semipermeable membrane (6.4 mm diameter and 0.4 µm pore size, Corning Costar, Corning, NY, USA) placed in 24-well plastic plates. A fresh culture medium with cells (0.5 mL) was added to the apical (AP) side and 1.5 mL of blank culture medium was added to the basolateral chamber. The medium in both the AP and BL sides was changed every 2–3 days. Monolayers were formed after culturing for 21 days. The integrity of the cell layer was evaluated by measurement of Transepithelial/transendothelial electrical resistance (TEER) with Millicell-ERS equipment (Millipore, MA, USA). Monolayers with TEER of >350 were used for the experiments.

To measure the apical-to-basolateral permeability, the culture medium was withdrawn and both the apical side of cell monolayers and the basolateral chambers were washed with modified Hank’s balanced salt solution (HBSS, Lowell, MA, USA). Then, 1.5 mL of HBSS (pH 7.4, 37 °C) was added to the basal chamber of the Transwell insert, and 0.5 mL of the samples (FE and L-FE) dissolved in HBSS (Lowell, MA, USA) were added to the apical side. After incubation at 37 °C, 0.5 mL of the basal solution was collected after 60 and 180 min. The intestinal absorption was determined by calculating the percentage of chlorogenic acid that passes through the membrane with respect to the initial amount of chlorogenic acid in each sample.

### 2.8. Determination of Phenolic Compounds

The profile of phenolic compounds in both FE and L-FE samples was determined before and after simulated gastrointestinal digestion (GID). In this regard, a middle step immediately after gastric digestion (GD) was also considered. The chlorogenic acid quantification was conducted in FE, L-FE, digest, and Caco-2 cells permeate samples. Analyses were performed by reverse-phase high-performance liquid chromatography (RP-HPLC, model SPE-MA10AVP, Shimadzu, Kyoto, Japan) on a C18 analytical column, following the procedure described in Alemán et al. [1].

### 2.9. Statistical Analysis

Each experiment was conducted in triplicate and the results were expressed as mean ± SD. Statistical significances were compared between each treated group and analysis of variance (ANOVA) was performed using SPSS Statistics 26 Software (IBM SPSS Statistics 22 Software, Inc., Chicago, IL, USA). The differences between means were assessed on the basis of confidence intervals by using the Tukey tests with the significance level set at *p* ≤ 0.05.

## 3. Results and Discussion

In a previous study, aqueous and ethanolic extracts of sea fennel, as well as liposomes of phosphatidylcholine (PC) from soybean lecithin loaded with these extracts, were already characterized [1]. Likewise, their antioxidant properties were evaluated, observing that ethanolic extracts showed higher antioxidant properties as expected but lower encapsulation efficiency. In order to delve into their biological properties, in the present work, choosing the aqueous extract for its higher encapsulation efficiency was considered more appropriate.

Firstly, the possible cytotoxicity of the extract (FE), the empty liposomes (L), and the encapsulated extract in the liposomes (L-FE) was evaluated, as well as their anti-inflammatory effect.

### 3.1. Cellular Viability

**Figure 1:**
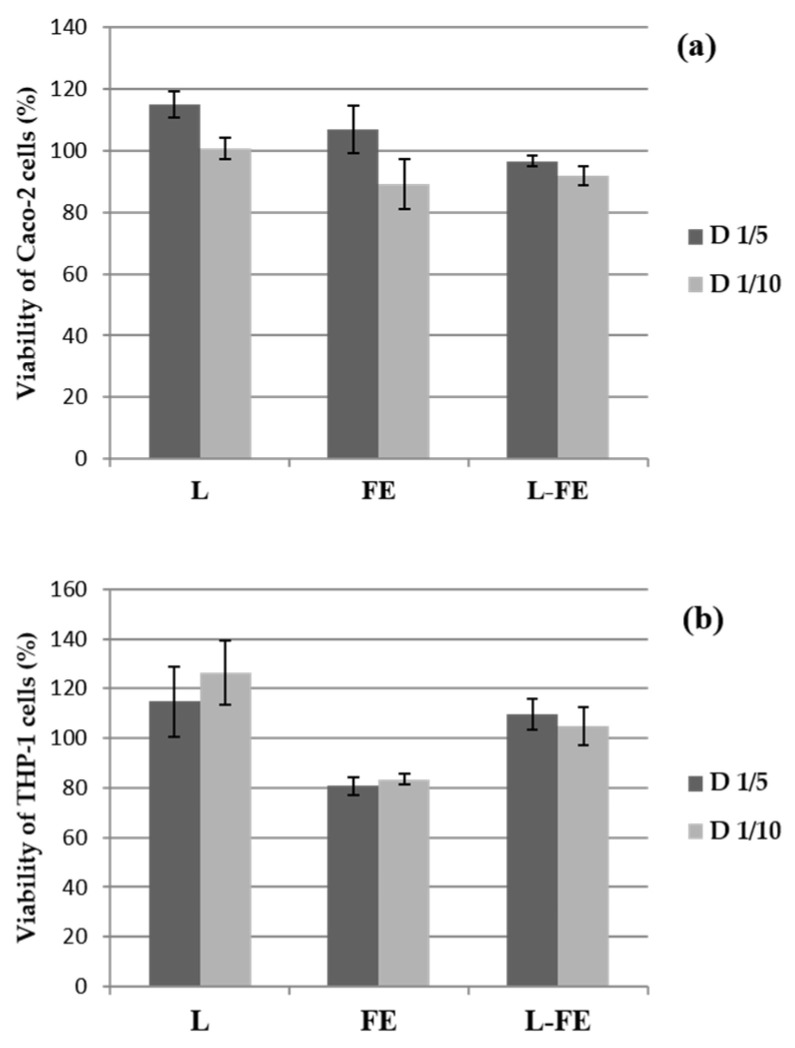
Percentage of viability of Caco-2 (**a**) and THP-1 cells (**b**) after incubation during 18 h with the samples at the two assayed concentrations: L (empty liposome), FE (sea fennel extract), and L-FE (liposome loaded with sea fennel extract). D 1/5 and D 1/10 indicate 1/5 and 1/10 dilutions of each sample, respectively (see Methods). Data show the mean ± S.D. (*n* = 3).

Cell viability of both THP-1 and Caco-2 cells evaluated after 18 h of incubation with the sea fennel aqueous extract (FE), the phosphatidylcholine liposomal dispersion (L), and the corresponding liposomes loaded with sea fennel extract (L-FE), is shown in Figure 1. The viability of the THP-1 cells against the tested samples was carried out as a prerequisite to evaluate their immune response, i.e., to assess their pro- or anti-inflammatory effect. Since the biocompatibility of the samples depends on both the biological properties and the concentration, the samples were tested at two different concentrations after incubation with THP-1 and Caco-2 cells, as mentioned above.

Cell viability was very high (Figure 1a,b) in all samples tested at both concentrations. Therefore, the samples were not cytotoxic since the percentage of viability was not below 80% in any of the cases. The increase in viability (approximately 120%) observed in some samples was also detected by Angius & Floris [19], who attributed this fact to inconsistent MTT absorbance values that do not correspond to a real increase in viability and are produced by interferences with sample compositions, for instance, with the lipid nature of liposomes. According to these authors, the chemicals and/or their conditions could affect the dehydrogenase activity in viable cells, which could cause a discrepancy between the actual number of viable cells and the number of cells determined by the Cell Counting Kit-8.

### 3.2. Anti-Inflammatory Activity on THP-1 Cells

The biocompatibility of the different samples (FE, L, and L-FE) in contact with THP-1 cells is a premise to study the immune response to verify the possible cytokine release that is induced by the action of these samples.

**Figure 2:**
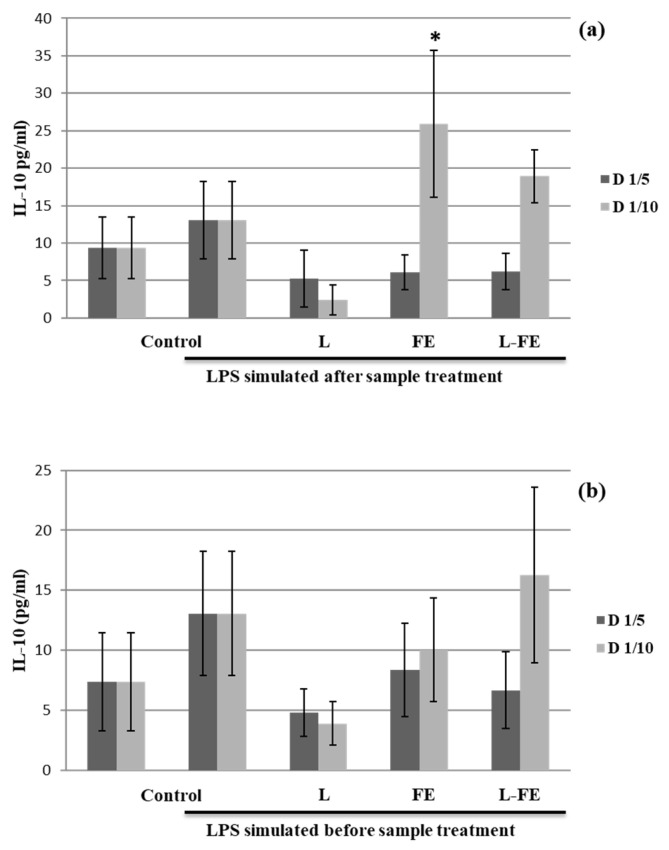
Effects of incubation with samples on IL-10 production in the THP-1 cells. The THP-1 cells differentiated to macrophages were incubated with no sample (Control), empty liposome (L), free fennel extract (FE), and liposome loaded with fennel extract (L-FE) for 18 h before (**a**) and after (**b**) treated with LPS for 4 h. D 1/5 and D 1/10 indicate 1/5 and 1/10 dilutions of each sample, respectively (see Methods). Data are expressed as mean ± S.D. (*n* = 3) for each dilution. * *p* ≤ 0.05 vs. LPS stimulated control cells by Tukey test.

**Figure 3:**
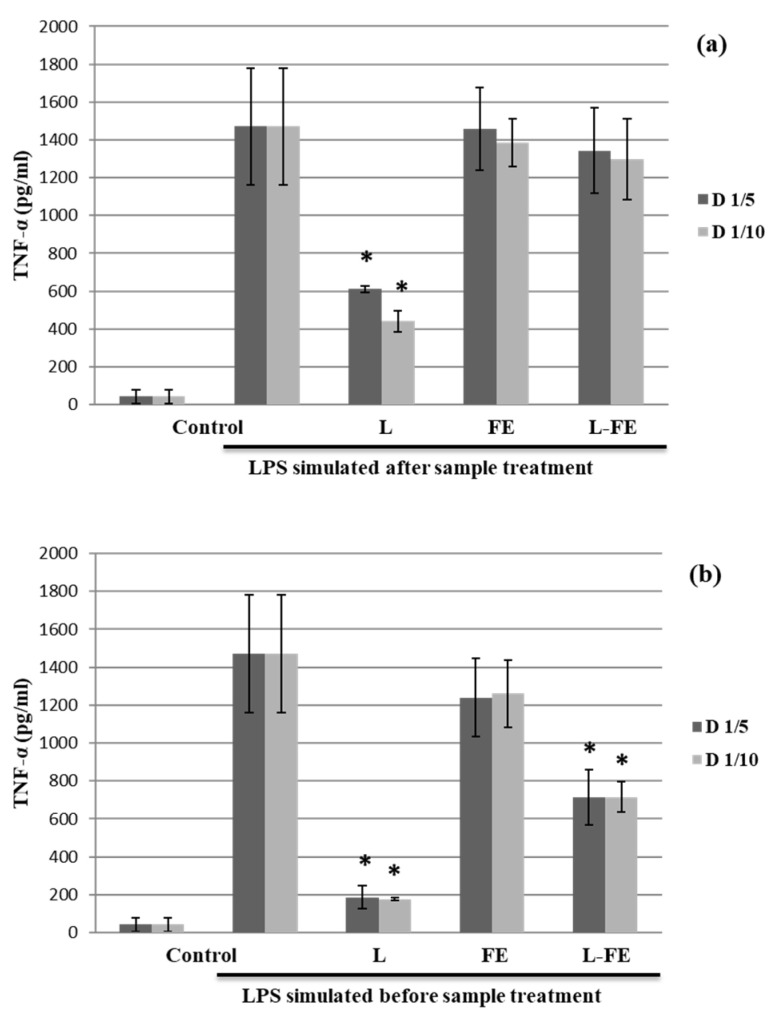
Effects of incubation with samples on TNF- α production in the THP-1 cells. The THP-1 cells differentiated to macrophages were incubated with no sample (Control), empty liposome (L), free fennel extract (FE), and liposome loaded with fennel extract (L-FE) for 18 h before (**a**) and after (**b**) treated with LPS for 4 h. D 1/5 and D 1/10 indicate 1/5 and 1/10 dilutions of each sample, respectively (see Methods). Data are expressed as mean ± S.D. (*n* = 3) for each dilution. * *p* ≤ 0.05 vs. LPS stimulated control cells by Tukey test.

For this study, THP-1 cells differentiated into macrophages were used in two assays to evaluate the anti- and pro-inflammatory effects of FE, L, and L-FE through the induced secretion of both IL-10 (Figure 2) and TNF-α cytokines (Figure 3). In a first assay, the THP-1 cells were first incubated with the samples and subsequently stimulated with LPS, to verify their protective anti-inflammatory activity. In parallel, a second assay, in which macrophages were initially stimulated with LPS and then exposed to the studied samples, was carried out to investigate their ability to downregulate the inflammation response, as previously described by De Marco et al. [8].

The data obtained from our study showed that only the supernatant recovered from the THP-1 cells incubated with the sea fennel aqueous extract (FE) at lower concentration (320 μg/mL, D1/10) and before LPS stimulation (assay 1) significantly induced Il-10 secretion (Figure 2a). However, FE could contain some components able to inhibit IL-10 production at high concentrations. Nevertheless, when macrophages are in an inflammatory state induced by pretreatment with LPS (assay 2), no induction of IL-10 was noted in any of the samples tested (Figure 2b). Therefore, the extract could be considered as an anti-inflammatory agent in a preventive way; however, once inflammation occurs, it does not show any effect on IL-10 secretion.

On the other hand, a decrease in TNF-α production was observed for the empty liposomes (L) in the THP-1 supernatants that were first pretreated with the samples and then stimulated with LPS (assay 1) (Figure 3a). In addition, when macrophages in an inflammatory state (induced by LPS) were later incubated with the samples (assay 2), both L and L-FE were able to downregulate the inflammation response by reducing significantly TNF-α secretion (at both concentrations tested), being the effect greater for L than for L-FE (Figure 3b). Therefore, the presence of sea fennel extract appears negatively affect the inhibition of TNF-α production caused by liposomes, maybe because the amount of liposome is lower in L-FE than in L, due to the dilution effect.

Considering these results (Figure 3), empty liposomes have a preventive effect and are also able of reducing inflammation once it occurs. In contrast, L-FE can only downregulate the inflammation once it occurs, but cannot be used to prevent inflammation.

Diverse authors have used THP-1 cells as an in vitro model for human cells (monocytes and macrophages) in studies on the mechanism of inflammatory diseases [20]. Also, another model proposed in the study of food compounds is based on the use of THP-1 cells as possible modulators of inflammation [21]; THP-1 cells were differentiated into macrophages to test the impact that certain compounds present in food can have on the immune system. This is based on the inflammatory reaction produced as a defense mechanism against foreign bodies or stimuli in the organism [22], macrophages having a fundamental role in this inflammatory reaction.

Regarding the anti-inflammatory activity of liposomes, it has been reported that liposomes from squid skin phospholipids have manifested anti-inflammatory effects, suggesting that they could act as apoptotic mimetics that lead to anti-inflammatory effects [23]. Plangsombat et al. [24], working with liposomes of phosphatidylcholine and cholesterol (ratio 7:3), found some anti-inflammatory activity, although this was greater when asparagus extract was encapsulated, the conditions being more effective at concentrations of 1:5 (extract: liposome)**.** According to Lordan et al. [7], phospholipids could contribute to several inflammatory cascades, including as inflammation mediators both PUFAS, arachidonic acid, oxidized phospholipids, and other fatty acids.

### 3.3. Effect of In Vitro Gastrointestinal Digestion

**Figure 4:**
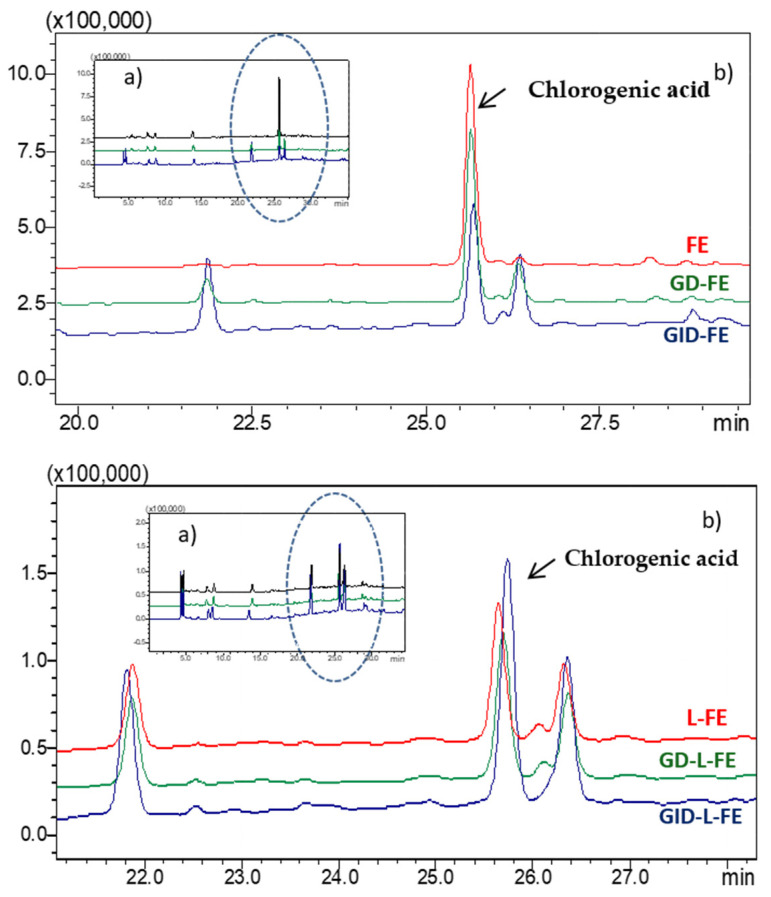
Chromatographic profile at λ 253 nm of the fennel extract (FE) and liposomes loaded with the extract (L-FE), before and after gastric digestion (GD) and gastrointestinal digestion (GID). (**a**) full chromatogram, (**b**) detail of the chromatogram with the most significant changes.

To determine the bioaccessibility of the sea fennel aqueous extract not encapsulated and encapsulated in liposomes (FE and L-FE), both samples were subjected to a GID process. At the end of the gastric phase (GD, 2 h with pepsin 4% *w*/*w* at pH 2, 37 °C), the chromatographic profile did not change significantly, showing a slight decrease (less than 5%) in the amount of chlorogenic acid in the extract, without any considerable modification in the liposome profile (Figure 4). Some studies have attributed the chlorogenic acid stability during gastric digestion to the acid pH of the medium, which provides a more suitable environment for the chemical stability of this compound [25].

On the other hand, at the end of the intestinal phase (GID, 2 h with bile salts and pancreatin 4% *w*/*w* at pH 7.2, 37 °C), chlorogenic acid was much more sensitive to degradation/transformation, and the amount present in the extract decreased by 40% (Figure 4, Table 1). The chromatographic profiles also showed an increase and/or the appearance of some unidentified compounds, which could be the result of the transformation of chlorogenic acid or other phenolic compounds present in the extract, as a consequence of enzymatic action, temperature, and/or medium pH. In a previous work of our group, splitting or transformation of chlorogenic acid was also observed when the sea fennel extract was encapsulated and was attributed to the heat effect due to sonication when forming the liposomes [1]. The new compounds formed could correspond to the isomers neochlorogenic or cryptochlorogenic acids [25,26]. Also, chlorogenic acid does not appear to be hydrolyzed, since among the new compounds formed, caffeic acid, which is a hydrolysis product, was not identified. With respect to the anti-inflammatory activity, TNF-α release has been previously reported to be inhibited by chlorogenic acid isomers, with cryptochlorogenic acid exhibiting the strongest effect [27]. The presumptively newly formed isomers in the digested sea fennel extract could also have improved the anti-inflammatory activity.

Previous studies suggest that the enzymes present in the small intestine do not usually influence the transformation of chlorogenic acid and that, however, it is the temperature, and especially the alkaline pH of the medium, that is mainly responsible for the decrease/transformation of this compound [28]. These authors found a 43–45% decrease in the content of chlorogenic acid after the gastrointestinal digestion of drinks prepared from yerba mate and coffee. Sengul et al. [29], also found that chlorogenic acid, present in a pomegranate extract, was stable during the gastric phase, but suffered a strong degradation at the end of the gastrointestinal digestion process, quantifying, in this case, a loss of 77%.

Conversely, Siracusa et al. [2], studying the effect of gastrointestinal digestion on a sea fennel infusion, found that chlorogenic acid was unstable in both the gastric and intestinal phases, with a final loss of 82% of the phenolic compound. Other authors, however, have observed high stability throughout the process of in vitro digestion of chlorogenic acid present in food matrices, such as edible flowers and fruit seeds [30,31,32].

In this work, the amount of chlorogenic acid quantified in the liposomes (L-FE) was only 33% with respect to the amount of chlorogenic acid in the extract (FE) and might correspond to the non-encapsulated part that remains free in the liposomal dispersion [1]. At the end of the digestion process of the liposomes, and contrary to what happens with the extract, the amount of chlorogenic was higher than in the undigested liposome dispersion. Thus, part of the chlorogenic acid that remained protected in the liposomes could have been released during GID, improving its bioaccessibility in the liposomal dispersion. The increase of the phenolic compound in the digested liposomes reached similar values (*p* ≤ 0.05) to those obtained for the digested extract (Table 1), so the amount of the free bioaccessible chlorogenic acid, at first, was similar for the extract and the liposome samples; however, it must be considered that a certain amount of chlorogenic could remain trapped inside the liposomes since according to different authors they partially resist the digestion process [33,34]. Machado et al. [33], observed that liposomes consisting of a mixture of soy lecithin and rice filled with spirulina polyphenols, were partially hydrolyzed during intestinal digestion, thus protecting the phenolic compounds they carried inside from gastric digestion, releasing them into the small intestine (jejunum and ileum). On the other hand, as mentioned above, during gastrointestinal digestion the chlorogenic acid released from the liposomes may also undergo transformation processes. Marín-Peñalver et al. [34], observed how the bioaccessibility of the active compound increased after a simulated GID in the case of soy phosphatidylcholine liposomes loaded with protein hydrolysate, suggesting it is due to a partial disintegration of the liposomes.

### 3.4. Intestinal Absorption

To evaluate the bioavailability of chlorogenic acid present in the sea fennel extract, both the extract (FE) and the liposomal dispersion (L-FE) were analyzed before and after being subjected to the simulated gastrointestinal digestion process. A well-established model of absorption, which consisted of bicameral permeable supports (Transwell) where Caco-2 cells were seeded and incubated until the formation of a differentiated monolayer and thus, emulated the passage of the extract through the small intestine, was used.

As can be seen in Table 2, the amount of chlorogenic acid that passed through the Caco-2 cell monolayer increased over time, although the difference was found to be significant (*p* ≤ 0.05) only for the free extract. However, the in vitro permeability of the phenolic compound, in general, was limited, being the content in the basolateral compartment only 1.4–1.6% of the initial amount after 3 h of incubation, without finding significant differences regardless of the encapsulation of the extract. The amount of chlorogenic acid passing through the membrane was therefore much lower than the free amount present in both the extract and the liposomal dispersion (non-encapsulated free fraction). In similar studies with coffee extracts, other authors have also found a low absorption of chlorogenic acid. Thus, Konishi & Kobayashi [11] and Dupas et al. [35], quantified values of less than 1% and 1.5% of the chlorogenic acid initially analyzed in the basolateral compartment, while Gómez-Juaristi [13] managed to recover 2.8% of the chlorogenic acid in the basolateral compartment.

Although several studies have shown that liposomes can cross the intestinal membrane and be absorbed rather easily [36], in the present work we did not detect the presence of liposomes in the basolateral compartment. In this same ex-vivo system [37], observed an increase in intestinal absorption for liposomes of soy lecithin and cholesterol loaded with a hexapeptide with ACE properties, much higher than that of the free peptide, so these authors indicated that the liposomes elaborated under these conditions are absorbable by the intestinal membrane. Using this same type of liposome formulation (soy phosphatidylcholine with cholesterol) but encapsulating chlorogenic acid, Feng et al. [15], conducted an in vivo study in mice, indicating that the encapsulation of chlorogenic acid increased 1.29 times the bioavailability of the phenolic compound and improved its antioxidant effect. Hence, the nature of the liposome may be important for its intestinal absorption capacity.

Different studies described a preferential absorption mechanism for chlorogenic acid, suggesting that transport would preferably occur in an apical-basolateral direction [11,12,13]. A great difficulty in this type of in vitro study is the instability of phenolic compounds under incubation conditions. Analyzing the content of chlorogenic acid in both the basolateral and apical compartments, we found that after 3 h of incubation, only 66–68% of the initial compound was recovered. Gómez-Juaristi [13] recovered a similar percentage of chlorogenic acid to that of this work (70%), while Scherbl et al. [38], observed an even greater degradation with a loss of up to 60% after 4 h of incubation. It must be considered that chlorogenic acid has been determined as the major compound of sea fennel extract, yet the determination of other compounds in the extract, or produced metabolites that could be absorbed, has not been addressed.

In human studies, it was found that, after the intake of 200 mL of coffee (20 mg of chlorogenic acids), the maximum concentration of unmetabolized chlorogenic acid in plasma was 2.2 nM, while other derivative metabolites (caffeic acid-3-O-sulfate and the sulfated lactones of 3-caffeoylquinic and 4-caffeoylquinic acids) reached maximum concentration values of 20–92 nM [39]. The results of the present work also suggest that most of the chlorogenic acid present in the sea fennel extract was not absorbed in the small intestine, and could reach the colon, where it would be susceptible to the action of the colonic microbiota, thus taking place the formation of microbial metabolites that could be subsequently absorbed, and/or presenting a possible prebiotic effect, as described by Jaquet et al. [40], for an extract of coffee rich in chlorogenic acid. According to Rechner et al. [41], chlorogenic acid, through two different transformation routes, with intermediate metabolites that can be absorbed by the large intestine or by the colon, could give rise to 3-hydroxyhypuric or hippuric acid, respectively, in the last transformation in the liver. Future studies are needed to assess whether liposomes, and the chlorogenic acid encapsulated in them, could reach the colon and be equally transformed by the colonic microbiota.

## 4. Conclusions

In conclusion, this work shows that the food components used (aqueous sea fennel extract and soy phosphatidylcholine liposome) had the in vitro capacity to decrease the inflammation response on THP-1 cells, being the effect of liposomes on TNF-α (proinflammatory) especially significant, while that of the sea fennel extract on IL-10 (anti-inflammatory effect) was less evident. Further studies, such as gene-expression analysis and the identification of mechanisms associated with anti-inflammatory activities, would be required to amplify the knowledge, given their possible use to improve human intestinal inflammation.

The sea fennel extract was susceptible to degradation/transformation during the gastrointestinal digestion process, with a decrease in the chlorogenic acid content of 40%. Part of the chlorogenic acid encapsulated in the liposomes was released during the intestinal phase of digestion so that at the end of the in vitro GID the amount of free bioaccessible chlorogenic was similar in the extract and in the liposomal dispersion. However, in the liposomal dispersion, a large amount of the extract could remain inside the liposomes. The intestinal absorption of chlorogenic acid, studied through a monolayer of Caco-2 cells, is limited, so that most of the free chlorogenic acid, as well as that which remains encapsulated in the liposomes, should reach the colon. Future studies are necessary to determine the transformation and the effect of liposomes in the colonic microbiota.

## Figures and Tables

**Table 1:** Chlorogenic acid concentration (μg/mL) in the sea fennel extract (FE) and the liposomes loaded with the extract (L-FE), before and after the in vitro gastric (GD) and gastrointestinal (GID) digestion.

	Chlorogenic Acid (μg/mL)
FE	40.3 ± 0.3 ^c^
L-FE	13.4 ± 0.8 ^a^
FE-GD	24.2 ± 0.3 ^b^
L-FE-GID	24.6 ± 0.5 ^b^

Data show the mean ± S.D. (*n* = 3). Different letters (a,b,c) indicate significant differences (*p* ≤ 0.05).

**Table 2:** Chlorogenic acid (%) that passed through the CACO-2 cell monolayer after 1 and 3 h of incubation.

	% Chlorogenic Acid (1 h)	% Chlorogenic Acid (3 h)
FE	0.83 ± 0.09 ^a/x^	1.53 ± 0.30 ^a/y^
L-FE	0.86 ± 0.12 ^a/x^	1.58 ± 0.43 ^a/x^
FE-GID	0.91 ± 0.15 ^a/x^	1.42 ± 0.61 ^a/x^
LFE-GID	0.90 ± 0.08 ^a/x^	1.49 ± 0.52 ^a/x^

Data show the mean ± S.D. (*n* = 3). The same letter (a) indicates that there are not significant differences (*p* ≤ 0.05) among samples. Different letters (x,y) indicate significant differences (*p* ≤ 0.05) between 1 and 3 h for the same sample.

## Data Availability

All data relevant to the study are included in the article.
